# Current clinical practice in disabling and chronic migraine in the primary care setting: results from the European My-LIFE anamnesis survey

**DOI:** 10.1186/s12883-020-02014-6

**Published:** 2021-01-04

**Authors:** Philippe Ryvlin, Kirill Skorobogatykh, Andrea Negro, Rainel Sanchez- De La Rosa, Heike Israel-Willner, Christina Sundal, E. Anne MacGregor, Angel L. Guerrero

**Affiliations:** 1 Department of Clinical Neurosciences, CHUV, Lausanne, Switzerland; 2 University Headache Clinic, Moscow, Russia; 3 Regional Referral Headache Centre, Sant’Andrea Hospital, Rome, Italy; 4 Department of Clinical and Molecular Medicine, Sapienza University, Rome, Italy; 5 Novartis Pharma AG, Region Europe Medical Dpt, Basel, Switzerland; 6 Specialized Center of Neurology Berlin (NFZB), Berlin, Germany; 7 Department of Neurology, Neuroclinic Norway, Lillestrøm, Norway; 8 Department of Clinical Neuroscience, Institute of Neuroscience and Physiology, The Sahlgrenska Academy, University of Gothenburg, Gothenburg, Sweden; 9 Barts Health NHS Trust, London, UK; 10 Centre for Neuroscience, Surgery and Trauma, Blizard Institute, Queen Mary University of London, London, UK; 11 Headache Unit, Neurology Department, Hospital Clínico Universitario, Valladolid, Spain; 12 Department of Medicine, University of Valladolid, Valladolid, Spain; 13 Institute for biomedical research of Salamanca (IBSAL), Salamanca, Spain

**Keywords:** Headache disorders, Chronic migraine, Anamnesis, Diagnosis, Management, Primary care, Guidance, Referral, Prophylaxis

## Abstract

**Background:**

Migraine is a prevalent and disabling headache disorder that affects more than 1.04 billion individuals world-wide. It can result in reduction in quality of life, increased disability, and high socio-economic burden. Nevertheless, and despite the availability of evidence-based national and international guidelines, the management of migraine patients often remains suboptimal, especially for chronic migraine (CM) patients.

**Methods:**

My-LIFE anamnesis project surveyed 201 General practitioners (GPs) from 5 European countries (France, Germany, Italy, Spain, and the UK) with the aim of understanding chronic migraine (CM) patients’ management in the primary care setting.

**Results:**

In our survey, GPs diagnosed episodic migraine (EM) more often than CM (87% vs 61%, *p* < 0.001). We found that many CM patients were not properly managed or referred to specialists, in contrast to guidelines recommendations. The main tools used by primary-care physicians included clinical interview, anamnesis guide, and patient diary. Tools used at the first visit differed from those used at follow-up visits. Up to 82% of GPs reported being responsible for management of patients diagnosed with disabling or CM and did not refer them to a specialist. Even when the GP had reported referring CM patients to a specialist, 97% of them were responsible for their follow-up. Moreover, the treatment prescribed, both acute and preventive, was not in accordance with local and international recommendations. GPs reported that they evaluated the efficacy of the treatment prescribed mainly through patient perception, and the frequency of follow-up visits was not clearly established in the primary care setting. These results suggest that CM is underdiagnosed and undertreated; thereby its management is suboptimal in the primary care.

**Conclusions:**

There is a need of guidance in the primary care setting to both leverage the management of CM patients and earlier referral to specialists, when appropriate.

**Supplementary Information:**

The online version contains supplementary material available at 10.1186/s12883-020-02014-6.

## Background

Headache disorders are a notable public health concern and a significant cause of disability worldwide. Among headache disorders, migraine is the main cause of recurrent headache (> 90%) [1], affecting 80.8 million individuals in Western Europe, and more than 1.04 billion world-wide [2]. Migraine impacts personal life but also has significant socio-economic and public health-related implications [3]. The Global Burden of Diseases, Injuries, and Risk Factors (GBD) study reported that migraine was responsible for 5.6% of all years lived with disability (YLDs) in the world, and for 6.2% of YLDs in Western Europe, and consider it to be the leading cause of disability in people 15–49 years old, accounting for 8.2% of YLDs worldwide [4, 5]. Between 2.5–3.1% of people with episodic migraine (EM) develop chronic migraine (CM) within one year [6–8], which is defined by headache on ≥15 days per month for ≥3 months, of which at least 8 days/month have migraine headache features [9, 10]. CM is associated with substantially greater reduction in quality of life and increased disability compared to EM, resulting in even greater personal and socio-economic burden [11–13].

Despite being highly prevalent and having significant personal and social impact, migraine may not receive appropriate attention, probably because it is not a life-threatening disease and may result in invisible disability and is common among the population. As a result, migraine and particularly CM, is largely underdiagnosed and undertreated worldwide [14, 15].

Migraine is a clinical diagnosis with primarily subjective manifestations and there are no diagnostic tests either for this condition or any of the primary headache disorders, nor for secondary headache disorders such as medication overuse headache (MOH). Thus, medical history and anamnesis are crucial for proper diagnosis [16]. Together with the medical history, the anamnesis done by asking specific questions to the patient will provide the physician with useful information to formulate the diagnosis. According to the International Classification of Headache Disorders (ICHD) and European Headache Federation (EHF), EM can be managed in primary care, but CM requires specialist referral because diagnosis and, particularly, management can be difficult [9, 17]. Nevertheless, in the Eurolight study carried out in 10 European countries, 33.8% of patients reported frequent migraine (> 5 days/month), of which < 18% had seen a GP, and < 15% had visited a neurologist [15]. Moreover, although 1/3 of patients reported frequent migraine and, therefore, need of preventive medication, < 11% of patients were receiving adequate acute treatment, and even a lower proportion (< 6.4%) were receiving preventive medication [15]. An observational study on the use of antimigraine treatments by French GPs identified that acute headache treatment is prescribed according to national practice guidelines and is considered as effective and satisfactory. In contrast, it showed that the use of preventive medication is low [18].

Considering the low proportion of people consulting GP and migraine specialists, and the mishandling reported of the acute and preventive treatments, it is crucial to better understand management of CM patients in the primary care setting. Thus, the aim of the My-LIFE anamnesis project was to describe the real current clinical practice regarding disabling and CM identification, treatment, and referral in the primary care setting in 5 European countries (France, Germany, Italy, Spain, and the UK).

## Methods

My-LIFE anamnesis project was designed as a survey to GPs from 5 European countries: France, Germany, Italy, Spain, and the UK using a structured online questionnaire.

The project involved two sets of participants: members of the Steering Committee (SC), and survey participants. The Pan-European SC included 7 members, well known clinical experts in migraine from Germany, Italy, Norway, Russia, Spain, Switzerland, and the UK. Their roles included performing literature reviews, defining the key objectives and the main content of the online questionnaire, and data interpretation.

For the survey, GPs from 5 European countries (France, Germany, Italy, Spain, and the UK) were invited to participate. In order to secure that participants had some background in the diagnosis and management of headache disorders the following selection criteria were defined: i) with at least 2 years of experience in general practice; ii) who were seeing at least 5 patients suffering from headache disorders per week; iii) usually proceeded with the medical history of their patients with headache disorders; iv) had currently at least 1 patient suffering from episodic and/or CM.

Ethics Committee approval was not applicable in this survey because its objective was to understand chronic migraine patients’ management in the primary care setting. There was no need to collect any type of patient data or information, hence the approval of an Ethics Committee or patient informed consent was not required. All survey participants gave their written consent.

### Questionnaire

Development of the questionnaire was based on a literature search and on the clinical experience of the SC members. The 32-item questionnaire was divided in three main sections: Participants’ profile; Current clinical practice in disabling or CM identification and treatment; and Definition of a Migraine Anamnesis Guide (Additional file 1). The questionnaire was written in English and translated into local languages (French, German, Italian, and Spanish). It was administered to 201 GPs fulfilling the inclusion criteria between 14th January 2020 and 28th January 2020 through an online platform that ensured data anonymity and confidentiality as well as that all questions were answered in order to avoid any missing value.

### Statistical analysis

Categorical variables were expressed in terms of means and standard deviations (SD) or range, or number and percentage of responses. Comparative analysis among countries was carried out with Chi-square test or ANOVA test for categorical and continuous variables, respectively. Data analysis was performed with the SPSS version 22, and *p* < 0.05 was considered to be statistically significant.

## Results

### Participants’ profile

In the My-LIFE anamnesis project, 201 GPs from 5 European countries were included: 41 GPs from France, and 40 GPs each from Germany, Italy, Spain, and the United Kingdom. GPs had 24 (range 2–42) years of experience and each saw 202 patients per week, on average. Twelve percent of patients presented with headache disorders, and migraine was diagnosed in 38% of them. Among migraine patients, 66% presented with EM, and 34% with CM (Additional file 2).

### Headache diagnosis

When the participants were asked about who did the diagnosis of EM or CM of their patients, the proportion of diagnosis ascertained by GP versus specialist significantly differed between EM (87% GP vs 13% specialist) and CM (61% GP vs 39% specialist) (*p* < 0.001). For EM diagnosis, no differences between countries were found; however, for CM significant differences (*p* = 0.015) were observed, ranging from 81% in France to 45% in Italy (Table 1).

**Table 1:** Migraine and chronic migraine diagnosis

		Total (***n*** = 201) n (%)	France (***n*** = 41) n (%)	Germany (***n*** = 40) n (%)	Italy (n = 40) n (%)	Spain (n = 40) n (%)	UK (n = 40) n (%)	***p*** value
**MIGRAINE DIAGNOSIS**								
**Episodic migraine**	Done by GP	175 (87.1%)	38 (92.7%)	33 (82.5%)	30 (75.0%)	37 (92.5%)	37 (92.5%)	NS
	Done by a specialist	26 (12.9%)	3 (7.3%)	7 (17.5%)	10 (25.0%)	3 (7.5%)	3 (7.5%)	
**Chronic migraine**	Done by GP	123 (61.2%)	33 (80.5%)	21 (52.5%)	18 (45.0%)	26 (65.0%)	25 (62.5%)	< 0.05
	Done by a specialist	78 (38.8%)	8 (19.5%)	19 (47.5%)	22 (55.0%)	14 (35.0%)	15 (37.5%)	
**DIFFERENTIAL DIAGNOSIS**								
**TTH rule-out**	Always	122 (60.7%)	21 (51.2%)	23 (57.5%)	15 (37.5%)	31 (77.5%)	32 (80.0%)	< 0.05
	Sometimes	59 (29.3%)	11 (26.8%)	17 (42.5%)	15 (37.5%)	8 (20.0%)	8 (20.0%)	
	Never	20 (10.0%)	9 (22.0%)	0 (0.0%)	10 (25.0%)	1 (2.5%)	0 (0.0%)	
**MOH rule-out**	Always	115 (57.2%)	17 (41.5%)	25 (62.5%)	12 (30.0%)	27 (67.5%)	34 (85.0%)	< 0.05
	Sometimes	72 (35.8%)	17 (41.5%)	14 (35.0%)	23 (57.5%)	12 (30.0%)	6 (15.0%)	
	Never	14 (7.0%)	7 (17.0%)	1 (2.5%)	5 (12.5%)	1 (2.5%)	0 (0.0%)	

*MOH* medication-overuse headache, *NS* non-significant, *TTH* tension-type headache

Headache disorders to be managed at primary care include tension-type headache (TTH), MOH and migraine [9], and GPs were asked about the differential diagnosis of these conditions. During the anamnesis of a patient with a headache disorder, 61% of participants always considered TTH, and 57% always ruled out MOH, but significant differences were observed among countries: 80% of UK GPs and 78% of Spanish GPs always ruled out TTH vs. 38% of Italian GPs. Consideration of MOH varied from 85% in the UK to 30% in Italy (*p* < 0.001, for both comparisons) (Table 1).

There are several instruments available to aid primary-care physicians in both migraine diagnosis and management. In our survey, the tools used to assess patients with disabling and CM at the first visit differed from those used in the follow-up visits. At the first visit, all participants (100%) conducted a clinical interview (vs. 51% at follow-up visits, *p* < 0.001), and 46% of them used an anamnesis guide (a guide to support the physician during the diagnosis) to diagnose CM (vs. 26% at follow-up visits, p < 0.001). In contrast, patient diaries were more often used at follow-up visits (69% vs. 36% at the first visit, p < 0.001), and also imaging techniques (50% vs. 21% at the first visit, p < 0.001).

**Fig. 1:**
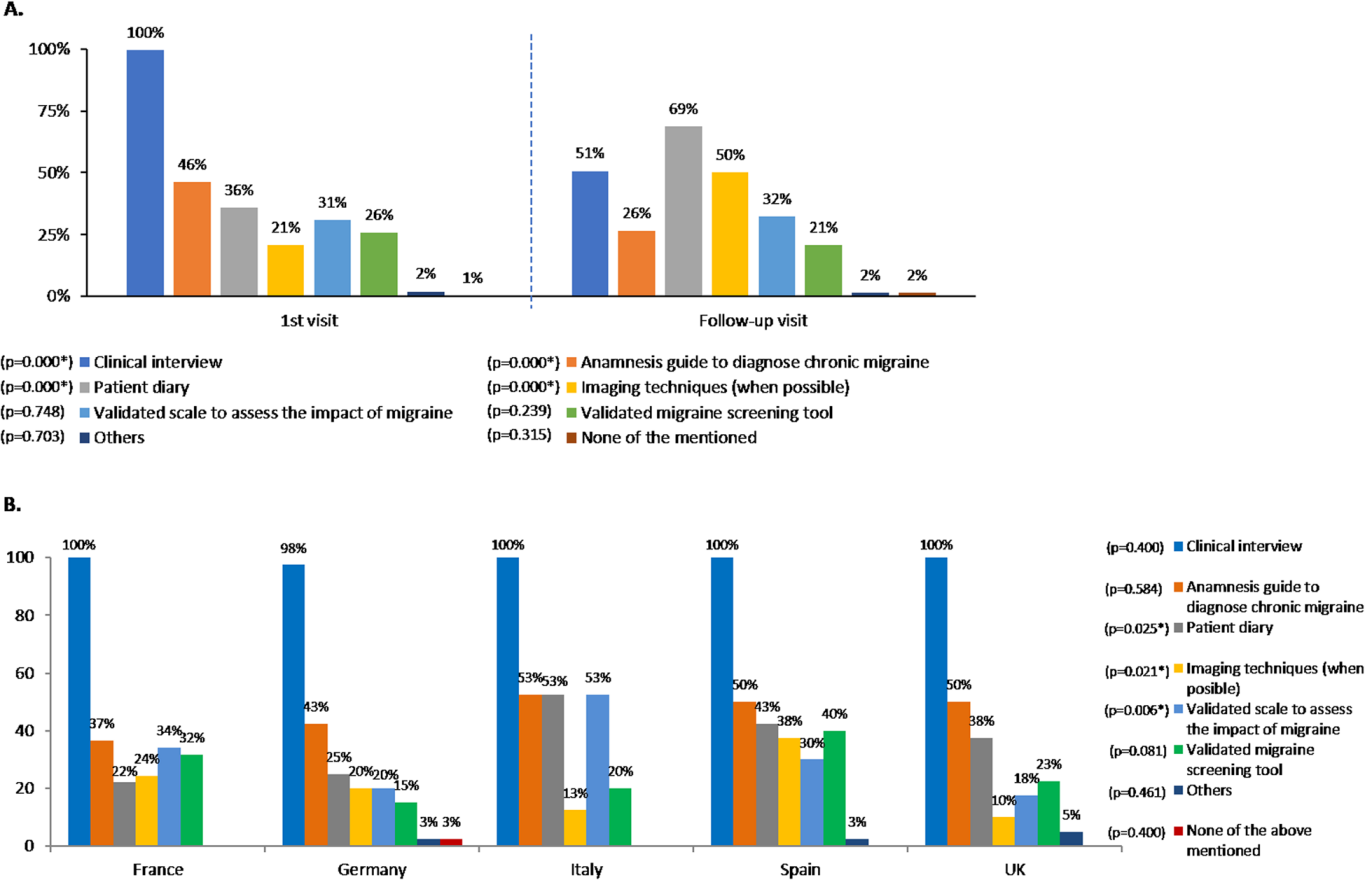
Tools used to assess patients with disabling or chronic migraine. **a**. Tools used at the first visit vs. tools used at the follow up visit (total). At the first visit,” others” included review of the medication and clinical examination including blood pressure; physical examination, and blood test. At the follow-up visit, “others” included physical examination, blood test, pain using a VAS, and patient information leaflets. *p < 0.05. **b**. Tools used at the first visit, by country. *p < 0.05

Validated scales to assess the impact of migraine and validated migraine screening tools were used by less than 35% of the participants at both the first and follow-up visits (Fig. 1a).

When considering the tools used by country in the first visit, significant differences were observed in patient diary use (*p* = 0.025), imaging techniques (*p* = 0.021), and validated scales to assess the impact of migraine (*p* = 0.006) (Fig. 1b).

### Anamnesis guide characteristics

Sixty-four percent of participants reported using an anamnesis guide to diagnose CM at any time. The guides used were heterogeneous, either developed by themselves (41%) or by the centre where the GP worked (15%), or validated and published guides (44%). Spain and Italy had the highest percentage of GPs using a validated and published anamnesis guide (54 and 52%, respectively) (*p* = 0.012) (Table 2).

**Table 2:** Characteristics of anamnesis guides used by GPs

ANAMNESIS GUIDE CHARACTERISTICS	Total (***n*** = 128) n (%)	France (***n*** = 25) n (%)	Germany (***n*** = 23) n (%)	Italy (***n*** = 27) n (%)	Spain (***n*** = 26) n (%)	UK (n = 27) n (%)	p value
**Anamnesis guide type**							< 0.050
Anamnesis guide validated and published (*n* = 56)	56 (43.8%)	8 (32.0%)	10 (43.5%)	14 (51.9%)	14 (53.8%)	10 (37.0%)	
Anamnesis guide developed by the centre where the responder works (*n* = 19)	19 (14.8%)	1 (4.0%)	1 (4.3%)	3 (11.1%)	8 (30.8%)	6 (22.2%)	
Guide/document developed by the respondent (*n* = 53)	53 (41.4%)	16 (64.0%)	12 (52.2%)	10 (37.0%)	4 (15.4%)	11 (40.7%)	
**Inclusion of red flags**	77 (60.2%)	12 (48.0%)	12 (52.2%)	5 (18.5%)	23 (88.5%)	25 (92.6%)	< 0.050

Inclusion of red flags i.e., signs or symptoms that can guide successive investigations and/or referral and decision-making, is common in primary care management of migraine. On average, 60% of the anamnesis guides used by GPs included red flags/warning features. Up to 93% of the anamnesis guides used in the UK and 89% in Spain included red flags vs. 19% in Italy (*p* < 0.001) (Table 2). There was no statistical difference between the anamnesis guide type used and the inclusion of red flags.

The main topics addressed during the anamnesis included frequency of attacks; medication use, frequency and effectiveness; and pain characteristics of the attacks, among others. These topics were independent of the anamnesis guide type used, and they were also independent of whether or not an anamnesis guide was used. However, when an anamnesis guide was not used, there was a trend to ask fewer questions and more differences between countries were found regarding the topics asked.

### Patient diary characteristics

**Fig. 2:**
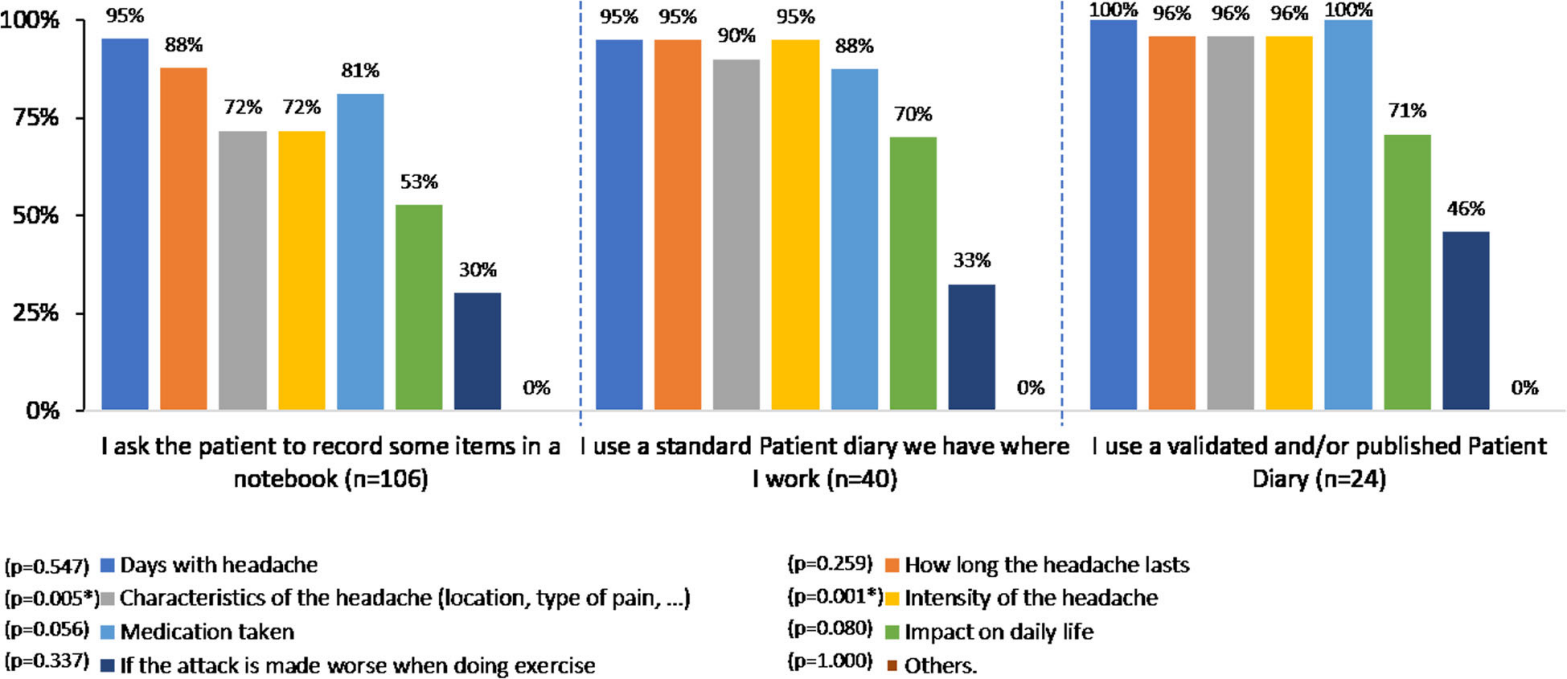
Main items recorded in the patient diary. *p < 0.05

Overall, 85% of participant GPs asked the patient to complete a patient diary, headache diary or calendar at any time. Most of the participants (62%) recommended recording some items on a notebook used as a patient diary instead of using a formal headache diary or calendar, and 38% recommended either a standard diary or a validated/published tool, without differences among countries. The main items requested to be recorded were frequency (96%) and duration (91%) of the attack, followed by medication taken and intensity of the headache (Fig. 2). It is noteworthy that, when using a validated/standard patient diary, information was more consistent and comprehensive, and more items were recorded, with statistically significant differences regarding the headache characteristics (*p* = 0.005) and intensity (*p* = 0.001).

### Other diagnostic tools

**Fig. 3:**
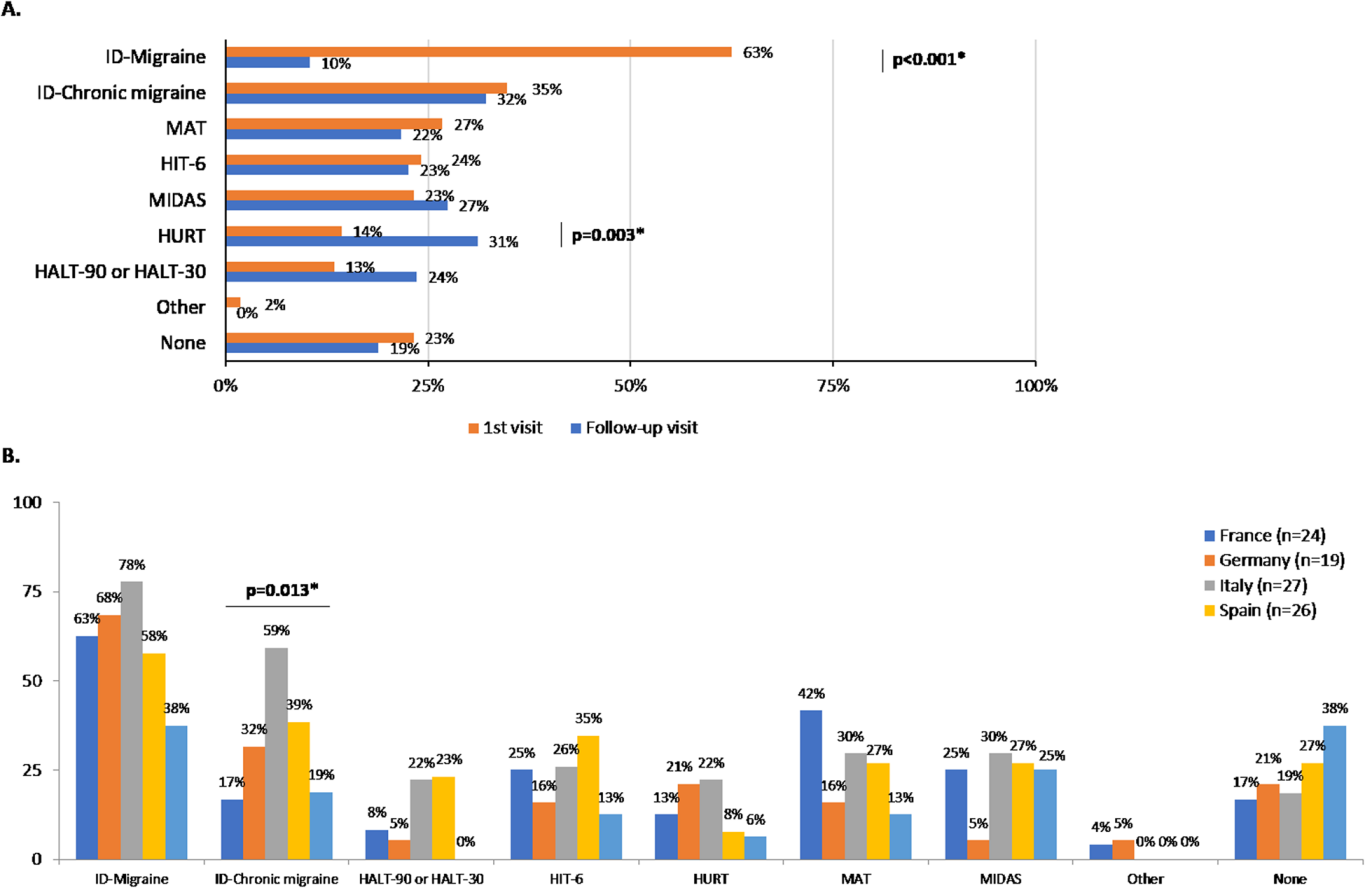
Validated scales used as diagnostic tools in patients with disabling or chronic migraine. **a** Validated scales used in the first visit vs. follow-up visit (total). *p < 0.050. **b**. Validated scales used by country in the first visit. *p < 0.050. †Other scales used with the patients with disabling or chronic migraine include KIEL HD questionnaire, Migraine Specific Quality of Life, and QVM score (Qualité de Vie et Migraine). HALT, Headache-Attributed Lost Time index. HIT-6, Headache Impact Test. HURT, Headache Under-Response to Treatment Questionnaire. MAT, Migraine Assessment Tool. MIDAS, Migraine Disability Assessment Score

Up to 50% of GPs used imaging techniques at follow-up visits to assess patients with disabling or CM (Fig. 1a). In this context, 58% of GPs stated that imaging techniques were mainly used to rule out secondary headache disorders. Besides, around 48% of the participants used validated scales to assess patients with migraine at any time. Among these, ID-Migraine was the most used in the first visit (63% of participants, *p* < 0.001) (Fig. 3a), while ID-Chronic migraine (ID-CM) (32%) and Headache Under-Response to Treatment (HURT) scales (31%, *p* = 0.003) were the most used validated scales in the follow-up visit. The ID-CM scale was employed by up to 59% of Italian GPs at the first visit (*p* = 0.013) (Fig. 3b).

### Chronic migraine management by GPs

**Fig. 4:**
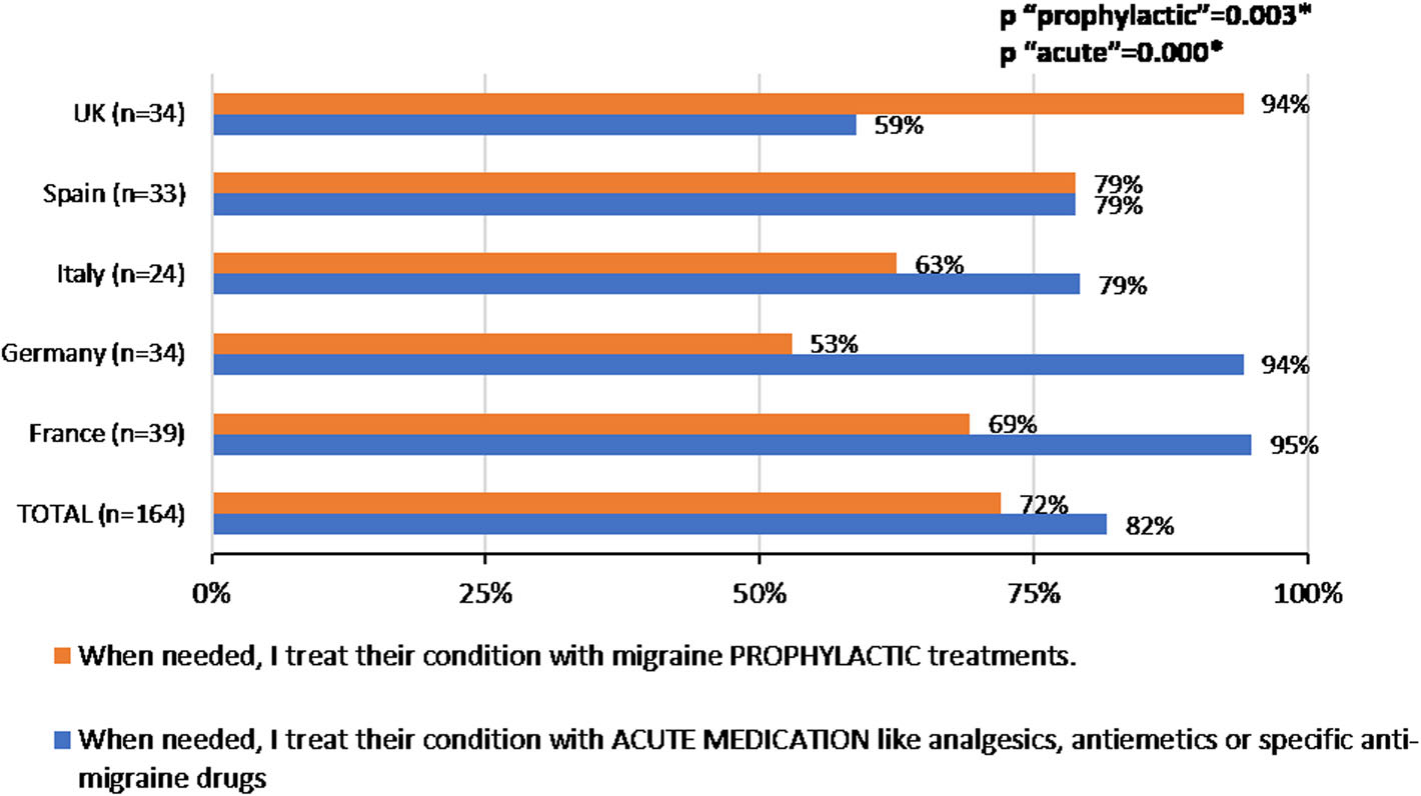
Treatments prescribed to disabling or CM patients by GPs, when not referring the patient

Overall, 82% of GPs reported that they usually managed patients diagnosed with disabling or CM themselves, without referral to a migraine specialist. Among these GPs, 82% reported prescription of acute medication and 72% of migraine preventive treatment when needed, with significant differences among countries for both acute (*p* < 0.001) and preventive treatment (*p* = 0.003). Up to 94% in Germany and 95% in France prescribed acute medication, and in the UK, 94% of the patients were prescribed preventive treatment (Fig. 4). There were no differences between countries, either in the percentage of patients prescribed acute medication prescribed or the percentage of patients prescribed preventive treatment.

Besides, GPs who reported that they were in charge of the treatment of CM patients considered that main reasons for referring patients include diagnostic uncertainty, lack of response to preventive or acute treatment, and or highly disabling migraine. Some differences were observed between countries: diagnostic uncertainty (*p* = 0.036), highly disabling migraine (*p* = 0.013), lack of response to preventive (*p* = 0.008) or acute treatment (*p* = 0.003).

### Chronic migraine patients’ referral and follow-up

**Fig. 5:**
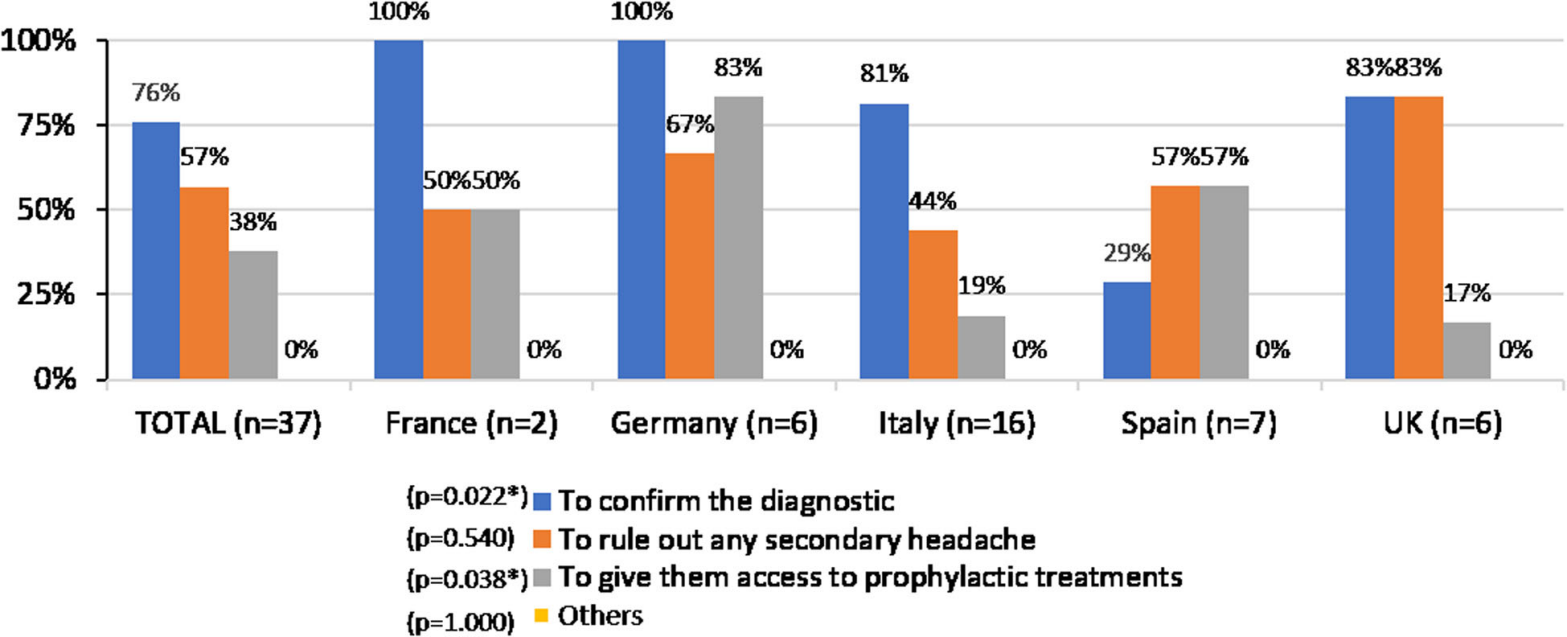
Reasons for referral of patients with disabling or chronic migraine, by country. *p < 0.050

Eighteen percent of the GPs reported that they “usually referred patients diagnosed with disabling or CM to a migraine specialist”. Among them, 76% mentioned that the main reason for referral was diagnostic confirmation (Fig. 5). In all countries 80–100% of GPs mentioned diagnostic confirmation as the main reason for referral, with the exception of only 29% in Spain (*p* = 0.022). In Germany, 83% of the participants referred their patients to give them access to a preventive treatment (*p* = 0.038); whereas preventive treatment access was not a strong reason for referral in Italy (19%) or in the UK (17%) (Fig. 5).

Even when the GP reported referring CM patients to a specialist for treatment prescription, up to 97% of GPs were in charge of ongoing management of most patients. The evaluation of the efficacy of the treatment prescribed by the specialists was mainly done through the patient perception (72%). Although non-significant, Spain was the only country where the ongoing evaluation was mainly done through a patient diary (86%). Moreover, the frequency of the treatment efficacy evaluation was not clearly established in the primary care setting, since yearly, semester, quarterly, monthly basis, and as needed evaluation were performed equally. To note, all French participants reported weekly evaluation of treatment efficacy (Table 3).

**Table 3:** Evaluation of the efficacy of the treatment when the patient is not referred

**ACUTE TREATMENT** (*n* = 134)	**Total (n = 134)** n (%)	**France (*n*** **= 37)** n (%)	**Germany (*n*** **= 32)** n (%)	**Italy (n = 19)** n (%)	**Spain (n = 26)** n (%)	**UK (n = 20)** n (%)	**p value**
**Evaluation of efficacy**							NS
Patient perception	100 (74.6%)	30 (81.1%)	23 (71.9%)	14 (73.7%)	17 (65.4%)	16 (80.0%)	
Patient diary	81 (60.4%)	21 (56.8%)	20 (62.5%)	12 (63.2%)	19 (73.1%)	9 (45.0%)	
Validated scale	16 (11.9%)	3 (8.1%)	4 (12.5%)	2 (10.5%)	23 (23.1%)	1 (5.0%)	
**Frequency of evaluation of efficacy**							NS
Weekly	16 (11.9%)	6 (16.2%)	5 (15.6%)	2 (10.5%)	1 (3.9%)	2 (10.0%)	
Monthly	67 (50.0%)	18 (48.7%)	12 (37.5%)	14 (73.7%)	13 (50.0%)	10 (50.0%)	
Quarterly	36 (26.9%)	12 (32.4%)	10 (31.3%)	3 (15.8%)	8 (30.8%)	3 (15.0%)	
Yearly	2 (1.5%)	0 (0.0%)	0 (0.0%)	0 (0.0%)	0 (0.0%)	2 (10.0%)	
When patient comes and complains	13 (9.7%)	1 (2.7%)	5 (15.6%)	0 (0.0%)	4 (15.3%)	3 (15.0%)	
**PREVENTIVE TREATMENT** (*n* = 118)	**Total (n = 118)** n (%)	**France (n = 27)** n (%)	**Germany (*n*** **= 18)** n (%)	**Italy (*n*** **= 15)** n (%)	**Spain (n = 26)** n (%)	**UK (n = 32)** n (%)	**p value**
**Evaluation of efficacy**							NS
Patient perception	89 (75.4%)	21 (77.7%)	12 (66.6%)	10 (66.6%)	19 (73.1%)	27 (84.4%)	
Patient diary	69 (58.5%)	17 (62.9%)	14 (77.7%)	10 (66.6%)	16 (61.5%)	12 (37.5%)	
Validated scale	15 (13.7%)	2 (7.4%)	2 (11.1%)	2 (13.3%)	6 (23.1%)	3 (9.4%)	
**Frequency of evaluation of efficacy**							< 0.050
Weekly	7 (5.9%)	6 (22.2%)	1 (5.5%)	0 (0%)	0 (0%)	0 (0%)	
Monthly	47 (39.8%)	9 (33.3%)	5 (27.8%)	8 (53.3%)	13 (50.0%)	12 (37.5%)	
Quarterly	48 (40.7%)	12 (44.5%)	9 (50.0%)	7 (46.7%)	9 (34.6%)	11 (34.4%)	
Yearly	4 (3.4%)	0 (0%)	0 (0%)	0 (0%)	0 (0%)	4 (12.5%)	
When patient comes and complains	12 (10.2%)	0 (0%)	3 (16.7%)	0 (0%)	4 (15.4%)	5 (15.6%)	

*NS* non-significant

In contrast, among GPs that usually treated CM patients themselves, the main indicators to evaluate the efficacy of acute and preventive treatments was the patient perception of the treatment (75%), and the frequency of the migraine attacks. On average, efficacy of the prescribed acute treatment was evaluated on a monthly basis by 50% of the participants. Of note, 10% of GPs evaluated the efficacy when the patient came back with complaints, without significant differences between countries (Table 3). The efficacy of the prescribed preventive treatment was evaluated on a monthly or quarterly basis by 81% of GPs on average. In the UK, Germany and Spain, 15–17% of GPs evaluated treatment efficacy when the patient came back complaining, while no GP reported this in Italy and France. In France up to 22% of GPs evaluated treatment efficacy on a weekly basis (*p* = 0.002) (Table 3). A small percentage of the participants used validated scales to evaluate the efficacy of treatment (acute or preventive) (Table 3); however, when the patient was referred and the GP was only in charge of follow up, GPs did not use validated scales.

## Discussion

The outcomes of this project provide data on the diagnosis and management of CM from 201 randomly selected GPs from 5 European countries, that could reflect what is currently being done in the primary setting with regards to HD disorders management. The main results from this survey suggest that many patients are underdiagnosed and undertreated. Most patients are not referred to specialists, and treatments prescribed, both acute and preventive, are not in accordance with local and international recommendations being the management of CM patients clearly suboptimal.

CM affects approximately 2% of the world population [19] and up to 4% of the European individuals [20]. Despite being an uncommon headache disorders, entails significant burden and should be recognized in primary care [9, 10], and this study indicates the need of increased awareness of CM. In the present survey, we found that 61% of CM diagnosis was made by GPs. Considering that diagnosis of migraine and CM is made solely through a proper anamnesis, the percentage of GPs that proceeded with a clinical interview in the follow-up visit (51%) is low. This may be explained by the limited time that can be dedicated to the patients in the primary care setting. According to guidelines [9], migraine diagnosis should include differential diagnosis of MOH and TTH, but only 61% of participants always ruled out TTH and 57% MOH. MOH requires an underlying primary headache disorder (most commonly migraine) and up to 2/3 of CM patients present with MOH [21]. The presence of MOH may complicate CM diagnosis, and even if MOH is concomitant with CM, it must be recognized and managed independently [9, 10, 17].

Proper management of migraine and other headache disorders requires continued monitoring of symptoms over time. For symptom monitoring in primary care, patient diaries are recommended because they can help to monitor headache intensity and frequency of symptoms [9, 10]. A positive finding from our survey was that almost 70% of participants reported the use of diaries in the follow-up visits.

In the follow-up visits imaging techniques were reported to be used by 50% of participants suggesting that CM patients are being submitted to imaging techniques too often, increasing the cost of CM patients management of the health care systems; however, current guidelines state that patients with headache and a normal neurological examination have the same the risk as the normal population of serious secondary pathology findings in neuroimaging [17, 22, 23]. Neuroimaging is not indicated unless the history or examination suggests headache may be secondary to another condition [9]; therefore, diagnostic uncertainty increases the use of unnecessary investigations, in agreement with previous studies [1, 24]. In addition, these results also confirm that CM is associated with a greater use of healthcare resources in the primary care setting [17].

Forty-six percent of participants used an anamnesis guide to diagnose CM. Whether a guide is used or not, awareness of specific warning features in the patient history to guide diagnosis and management is recommended [9, 10]. Besides, the inclusion of red flags in the anamnesis guide was independent of the type of guide; however, more than 85% of the anamnesis guides used by Spanish and UK GPs included these red flags. The proper use of red flags would help to identify those patients where imaging is really needed, but these results do not seem to be related with a decrease in the use of imaging techniques to rule out secondary headache disorders. Current guidelines suggest specific warning features/red flags that should be detected in the medical history for differential diagnosis of the headache disorders relevant to primary care [9]. Recently, Phu Do et al. developed the systematic SNNOOP10, a list of red and orange flags useful for detecting secondary headache disorders in clinical practice [24].

Effective management of migraine needs to consider the impact on the patient’s life and lifestyle to establish the best treatment. In primary care, up to 18 scales are used to classify or screen for migraine [25]. Although most of the tools are quick, easy-to-use, self-completed screening questionnaires, their use is not widely widespread. In this line, we observed that fewer than 50% of the participants used a validated scale as a screening tool or during the follow-up. Considering how disabling migraine is, it would be of high interest to enhance relevant outcomes measurement in clinical routine in these patients.

The most widely used tool at the first visit was ID-Migraine (63% of participants). This scale has been validated in different languages and settings and is common among GPs [25–28]. Interestingly, 59% of participants from Italy were using the ID-CM at the first visit, which perhaps could be explained by the recent translation and validation of this scale into Italian [29]. The ID-CM scale is a self-administered tool to help identify individuals with CM [14]; however, it does not identify other chronic headache disorders since it does not include either warning features to identify secondary headache disorders, or referral recommendations.

The most used scales by GPs during follow-up included ID-CM, MIDAS, and HURT. Despite not being experts in migraine, the percentage of GPs using the MIDAS or the HURT scales suggests that the GPs in this survey had experience in migraine management, as these scales are not so widespread among GPs.

The anamnesis relies mostly on patient perception of attack frequency and the evaluation of treatment efficacy, both acute and preventive, reinforcing the importance of patients reported outcomes for migraine management in the real-life setting [30, 31].

Considering that the prescription of some preventive treatments (onabotulinum toxin A and CGRP antibodies) for disabling and CM patients is restricted to migraine specialists in most European countries, the low referral rate reported would support the fact that the majority of CM patients seem not to receive adequate preventive treatment. The low use of preventive medication among migraine patients has been previously reported [15, 18]. In Germany, 83% of participants stated that the main reason for referring their patients to a specialist is to give them access to a preventive treatment. Moreover, preventive treatment would also reduce the risk of MOH development in CM patients [17]. Nowadays, a range of effective and well tolerated preventive drugs for CM exists. Patient access to preventive drugs remains a challenge in Europe and it is important that all healthcare professionals managing CM are well informed about all treatment options and the specific prescription conditions in their country.

European and local current guidelines recommend referral of patients with CM from primary care to specialist care because diagnosis and management can be difficult [9, 17, 32]. The low referral rate reported by the GPs could also be explained by a long wait for patients to see a specialist, thus giving more pressure on the primary care setting and increasing the visit to the emergency departments. The assessment of headache disorders patients in the emergency department setting differs from the assessment in primary care. A recent retrospective study analysed migraine management in the emergency room (ER) to identify deficiencies that could be solved by a rapid referral to a headache centre. The concordance analysis between ER diagnosis and tertiary level headache centre diagnosis showed a significant moderate agreement for the diagnosis of migraine between triage and headache centre. Most patients attending ER complaining of headache received the same treatment independently of their diagnosis; thus, rapid referral to a headache centre is key to provide a definite diagnosis and appropriate treatment [33]. In this line, the Spanish Society of Neurology’s Headache Study Group (GECSEN) has issued a series of recommendations constituting a referral protocol to guide decision-making in patients with headache. This protocol for action and referral from ER and primary care is aimed to improve diagnosis and treatment in patients with headache (both primary and secondary) and/or craniofacial neuralgia [34, 35].

Despite the GP participants having knowledge and experience of migraine management, the results from this survey clearly show that CM patients are undertreated. Considering that this group of GP’s sees on average 40 patients a day, it would be very challenging to provide specialist management of CM patients giving an additional explanation to the undertreatment of these patients. All this suggesting that in a less experienced primary care setting, the under-treatment of CM patients could be more extensive. The Vancouver Declaration on Global Headache Patient Advocacy agreed on the need of adequate training in Headache Medicine of all HCP and that all patients affected by headache disorders should have reliable access to competent medical care [36].

Finally, it should be noted that some country differences may also be explained by differences in the local healthcare system, the patient’s journey and locally available guidelines. Nevertheless, the main strength of our findings derives from the participation of GPs from several European countries providing detailed information regarding the clinical management of CM patients.

This study has some limitations. It is based on a survey and therefore relies on participants’ recall; thus, the risk of a selection bias of the participating GPs cannot be excluded. Moreover, GPs included had clinical experience with headache disorders suggesting that results regarding under-treatment of CM in primary care could be even higher than reported.

In summary, this project provides information on chronic and disabling migraine management in Europe and suggests the need for guidance in the primary care setting in order to leverage the diagnosis and treatment in such patients.

## Conclusions

Despite the availability of evidence-based national and international guidelines, our study emphasizes that there is a need to improve the diagnosis and management of migraine in the primary care setting and more specifically the diagnosis, management, and referral, when appropriate, of CM patients.

## Supplementary Information

**Additional file 1.** Online questionnaire. 32-item questionnaire run online among GPs.

**Additional file 2.** Profile of patients attended by GPs in 1 week, on average.

## Data Availability

The datasets used and/or analysed during the current study are available from the corresponding author on reasonable request.

## Abbreviations

CM: Chronic migraine
EHF: European Headache Federation
EM: Episodic migraine
GBD: Global Burden of Diseases, Injuries, and Risk Factors
GP: General practitioner
ICHD: International Classification of Headache Disorders
MOH: Medication-overuse headache
TTH: Tension-type headache
YLD: Years lived with disability
