# Supplementary figures and images for: Current clinical practice in disabling and chronic migraine in the primary care setting: results from the European My-LIFE anamnesis survey
Source: BMC Neurol. 2021 Jan 4;21:1. doi: 10.1186/s12883-020-02014-6 (PMC7780632; doi:10.1186/s12883-020-02014-6)

**Additional file 2.** Profile of patients attended by GPs in 1 week, on average


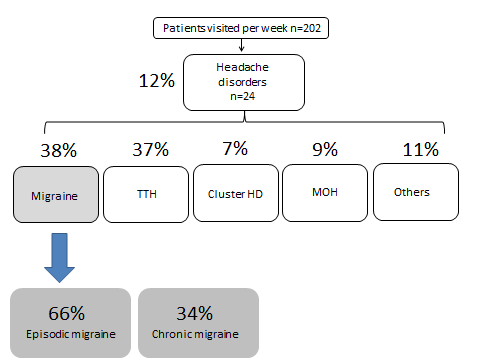

Supplement: Supplementary file 2 — Additional file 2. Profile of patients attended by GPs in 1 week, on average. [file 12883_2020_2014_MOESM2_ESM.docx]
